# Effect of Tai Chi on Markers of Oxidative Stress: Systematic Review and Meta-Analysis

**DOI:** 10.3390/ijerph18073458

**Published:** 2021-03-26

**Authors:** Juana Rosado-Pérez, Osvaldo D. Castelán-Martínez, Abril J. Mújica-Calderón, Martha A. Sánchez-Rodríguez, Víctor Manuel Mendoza-Núñez

**Affiliations:** 1 Research Unit on Gerontology, Facultad de Estudios Superiores Zaragoza, Universidad Nacional Autónoma de México, Ciudad de México P.C. 09230, Mexico; juanarosadoperez@comunidad.unam.mx (J.R.-P.); joseimujika@gmail.com (A.J.M.-C.); masanrod@comunidad.unam.mx (M.A.S.-R.); 2 Clinical Pharmacology Laboratory, Facultad de Estudios Superiores Zaragoza, Universidad Nacional Autónoma de México, Ciudad de México P.C. 09230, Mexico; castelan@unam.mx

**Keywords:** Tai Chi, oxidative stress, antioxidant enzymes, walking, yoga

## Abstract

Background: This study aimed to synthesize the evidence of the effect of practicing Tai Chi on oxidative stress markers (OxSM). Methods: This systematic review and meta-analysis was conducting using the MEDLINE, Cochrane Library, ScienceDirect, Scopus, Epistemonikos, Lilacs, and Ovid databases to identify randomized (RCT) and non-randomized (NRCT) clinical trials that evaluated the Tai Chi effect on OxSM compared to sedentary behavior, walking or yoga. Pooled mean differences (MDs) with 95% confidence intervals (95%CI) were estimated using the inverse variance method to determine the effect of Tai Chi on OxSM. PROSPERO register: CRD42019138362. Results: Five RCT and five NRCT were included. Compared to sedentary behavior, regular Tai Chi practice increases the levels of the enzymes superoxide dismutase (MD = 34.97 U/mL, (95%CI, 9.45 to 60.48), 344 participants) and catalase (MD = 15.63 U/mL, (95%CI, 4.05 to 27.22), 110 participants), as well as reducing the levels of lipoperoxides (MD = −0.02 µmol/L, (95%CI, −0.04 to −0.00), 234 participants). For comparisons with walking or yoga, only one study per activity was identified comparing the effect on OxSM. Conclusions: Regular Tai Chi practice increases the levels of superoxide dismutase and catalase, as well as reducing the levels of lipoperoxides. More studies are necessary to determine the effect of Tai Chi on OxSM when compared to other physical activities.

## 1. Introduction

Oxidative stress (OxS) is a biochemical alteration in which the imbalance between the production of reactive species (RS) and antioxidants, in favor of the former, promotes the oxidative damage of biomolecules and alterations in cellular physiology, and, although RS plays a very important physiological role, OxS has been linked to the development of many diseases and their complications, as well as with the aging process [1,2,3].

It has been shown that aging per se increases OxS as well as its association with various pathologies, such as neurodegenerative and cerebrovascular diseases, chronic renal diseases, diabetes mellitus 2, hypertension, atherosclerosis, and cancer. [3]. For this reason, different therapeutic alternatives have been proposed to control the OxS associated with the pathophysiology of some diseases, among which the consumption of melatonin, a diet rich in antioxidants, and moderate physical exercise can be highlighted [4,5,6,7].

In this sense, Tai Chi is a form of traditional Chinese exercise based on modifications to martial arts that is characterized by a series of smooth and continuous movements, whose execution requires position control, deep breathing, and coordination of the movements in the legs, arms, torso, and head to move from one position to another, moving the balance point without stopping. All this is done under a state of relaxation and concentration essential to achieving balance in movement. It is classified as moderate physical activity, given the maximum oxygen expenditure and heart rate involved [8,9,10].

Given its nature, it is greatly acceptance in the gerontological sphere, and in this sense, numerous studies report the benefits of Tai Chi in aspects such as balance, stability, and elasticity, which are essential elements to maintaining gait and decreasing falls. Improvements in the cardiovascular, respiratory, immune, and endocrine systems, and psychological responses, have also been reported, since the practice of Tai Chi requires deep concentration in addition to memorization of the movements, their names, and their sequence [8,9,10,11,12].

Several clinical trials have reported that the practice of Tai Chi has an antioxidant effect, which is attributed to a possible hormetic response due to the sustained stimulus, which involves a degree of physical effort, breathing, and transcendental meditation, characteristic of this activity [11,12]; however, this evidence has not been systematically analyzed. Thus, this study aimed to synthesize the evidence of the effect of practicing Tai Chi on OxS markers (OxSM).

## 2. Materials and Methods

This research was performed according to the preferred reporting items for systematic reviews and meta-analyses (PRISMA) guidelines, and the protocol was prospectively registered on PROSPERO (CRD42019138362).

### 2.1. Search Strategy

We performed a comprehensive literature search in the databases of MEDLINE, Cochrane Library, ScienceDirect, Scopus, Epistemonikos, Lilacs, and Ovid, from inception to 21 September 2019. In addition, a search was carried out to identify potential studies in ScienceDirect conferences, Scopus conferences, ProQuest, Tesis IPN and Tesis UNAM. The search strategy was designed to be as broad as possible and was constructed by combining the medical subject headings (MeSH) descriptors “Oxidative Stress” and “Tai Ji”, as well as the keyword “Tai Chi”. The strategy was adapted to each database. Searches were not limited by language or date. In the studies included, references were reviewed manually to identify studies that met the inclusion criteria.

### 2.2. Study Selection and Data Extraction

The eligible articles included in this study must meet the following criteria: (1) randomized (RCT) and non-randomized (NRCT) clinical trials; (2) the participants were adults with any clinical condition; (3) participants performed Tai Chi regularly and had done for at least two months; participants in the control group maintained a sedentary behavior or performed either walking or yoga; and (4) the result of the study was the quantification of OxSM. Studies wherein participants performed Tai Chi Chuan were excluded. Two reviewers (AJM and ODC) independently reviewed and assessed all titles and abstracts in order to identify studies via the inclusion criteria, and excluded non-relevant studies. The reviewers were blinded to each other’s decisions. Discrepancies were discussed and resolved with other reviewers (JR and MAS). 

An Excel database was used to extract the following information from the included studies: first author, publication year, study type, intervention, follow-up, participant’s characteristics, number of participants in each group, means and standard deviations of OxSM for each group. In studies that have more than one measurement point, the data from the last measurement point will be taken to be included in the meta-analysis. The reviewers (AJM, JR and ODC) independently reviewed and extracted data from the included studies and discrepancies were resolved by consensus. 

### 2.3. Risk of Bias Assessment

Potential biases related to individual randomized studies were assessed with the Cochrane risk of bias assessment tool [13]. RevMan 5.3 was employed to generate the risk of bias figure. The risk of bias for the non-randomized studies was assessed using the ROBIS-I tool [14]. Risk of bias was assessed in duplicates by two authors independently (AJM and ODC), with disagreements addressed by re-evaluation, in conjunction with a third reviewer (MAS).

### 2.4. Statistical Analysis

Statistical analyses were performed using RevMan 5.3 [15]. The means of each OxSM were analyzed using the DerSimonian and Laird method to obtain a pooled mean difference (MD) with 95% confidence intervals (95%CI). If necessary, standard deviations were calculated from the mean, standard error, and number of participants using GraphPad QuickCalcs (GraphPad Software, Inc, San Diego, CA, USA). Studies with insufficient information to be included in the meta-analysis were excluded. For the enzyme superoxide dismutase, a sub-analysis was performed in terms of Tai Chi sessions per week. For the heterogeneity assessment, I^2^ statistical analysis was performed. I^2^ values ≤25%, 25–50%, 50–75%, and >75% indicated no, small, moderate, and significant heterogeneity, respectively. Leave-one-out sensitivity analysis was utilized to determine if the pooled effects were robust, and the study with the greatest effect was excluded. A sensitivity analysis was conducted only when three or more studies were included in the comparison. Publication bias was assessed using the Egger regression asymmetry test when the comparison contained at least 10 studies. 

## 3. Results

### 3.1. Study Selection and Study Characteristics

**Figure 1:**
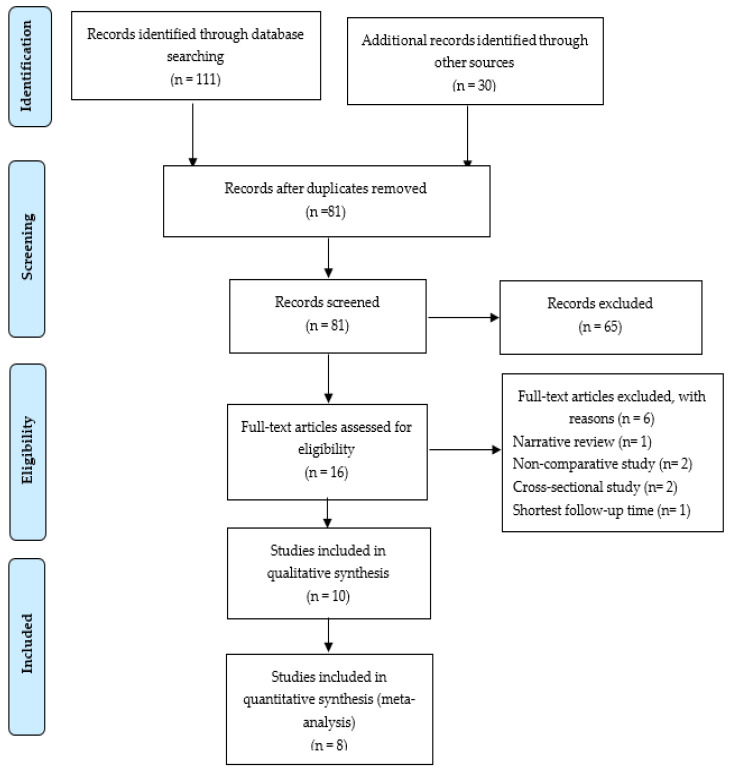
Preferred reporting items for systematic reviews and meta-analyses (PRISMA) flow diagram for article screening and selection process.

In the systematic search, a total of 141 citations were identified, and the study selection process is illustrated in Figure 1. After duplicate removal, 81 studies were assessed via their title and abstract for potential eligibility. In total, 65 studies were excluded, and 16 studies were retrieved for full text analysis. Finally, ten studies fulfilled the inclusion criteria of being a systematic review, and the characteristics of the included studies are summarized in Table 1. [16,17,18,19,20,21,22,23,24,25]. Reasons for the exclusion of the six studies [26,27,28,29,30,31] are shown in Supplementary Table S1.

Five RCTs [16,17,18,19,20] and five NRCTs [21,22,23,24,25] were included in this research. Eight studies evaluated the OxSM in blood [16,18,19,20,21,22,23,25], while one study determined the OxSM in saliva [24], and another in urine [17]. Two studies were excluded from the quantitative analysis, the first because it did not report the units in which it measured the OxSM [18], and the second because it did not report the means and standard deviations of the OxSM [21]. Finally, eight studies were included in the meta-analysis.

A total of 612 participants were included in the trials that evaluated the effect of Tai Chi practice on markers of oxidative stress, ranging in age from 18 to 74 years. Among all 612 patients, 286 practiced Tai Chi, 271 displayed sedentary behavior, 43 practiced walking and 12 practiced yoga.

### 3.2. Tai Chi Effect on Markers of Oxidative Stress

**Figure 2:**
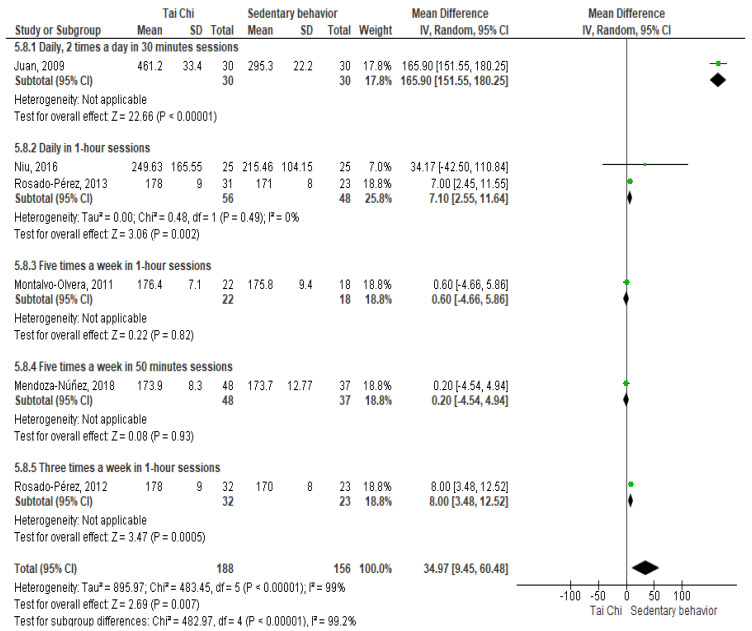
Forest plot of mean difference summary of the effect of Tai Chi on superoxide dismutase activity in comparison with sedentary behavior. The sub-analysis was performed according to the Tai Chi sessions per week.

Six studies evaluated the effect of Tai Chi practice on the enzyme superoxide dismutase (SOD) [16,19,20,22,23,25] (Figure 2). The results of the meta-analysis showed an increased activity of the enzyme SOD in the group that practiced Tai Chi compared to the participants who showed sedentary behavior (MD = 34.97 (95%CI 9.45, 60.48), 344 participants, significant heterogeneity). In the sensitivity analysis, the effect of Tai Chi on SOD was maintained after excluding Juan 2009 [16] (MD = 4.15 (95%CI 0.21, 8.10), 284 participants, moderate heterogeneity). In contrast, when SOD was determined in saliva, one study [24] showed no difference between participants who practiced Tai Chi and those who were sedentary (Table 2). Similarly, one study showed that there was no difference in SOD activity between participants who practiced Tai Chi and those who walked [23]. 

The pooled mean difference from six studies showed that there was no difference in glutathione peroxidase activity (GPx) between participants who performed Tai Chi and those who were sedentary [16,19,20,22,23,25] (Table 2). The result was consistent after performing the sensitivity analysis by excluding Niu 2016 [19] (MD = 1.20 (95%CI −1.46, 3.87), 294 participants, significant heterogeneity). In comparison with walking, the participants who practiced Tai Chi had lower glutathione peroxidase levels (Table 2).

Interestingly, the practice of Tai Chi reduced the levels of lipoperoxides (LPO) [20,22,23,25]. The robustness of this result was achieved in the sensitivity analysis by excluding Mendoza 2018 [25] (MD = −0.04 (95%CI −0.07, −0.01), 149 participants, no heterogeneity). Likewise, Tai Chi reduced LPO compared to walking [23] (Table 2). In the case of malondialdehyde (MDA), the pooled mean of two studies indicated a tendency to be lower in Tai Chi practitioners compared to sedentary participants [16,19].

The results of the meta-analysis show that the participants who practiced Tai Chi exhibited increased catalase activity compared to those who maintained a sedentary lifestyle. However, the effect was not observed when the determination was performed via saliva (Table 2).

Only one study evaluated the marker 8-hydroxy-2′-deoxyguanosine (8-OHdG) in urine [17]. The results suggest an 8-OHdG reduction in the patients who practiced Tai Chi compared to the sedentary participants.

In the studies included in this systematic review and meta-analysis, no differences were observed between Tai Chi and sedentary participants in the following markers: total antioxidant status (TAS), glutathione, saliva TAS and saliva lipoperoxides. Similarly, no difference was observed in TAS between the Tai Chi-practicing and walking participants (Table 2).

### 3.3. Quality Assessment

**Figure 3:**
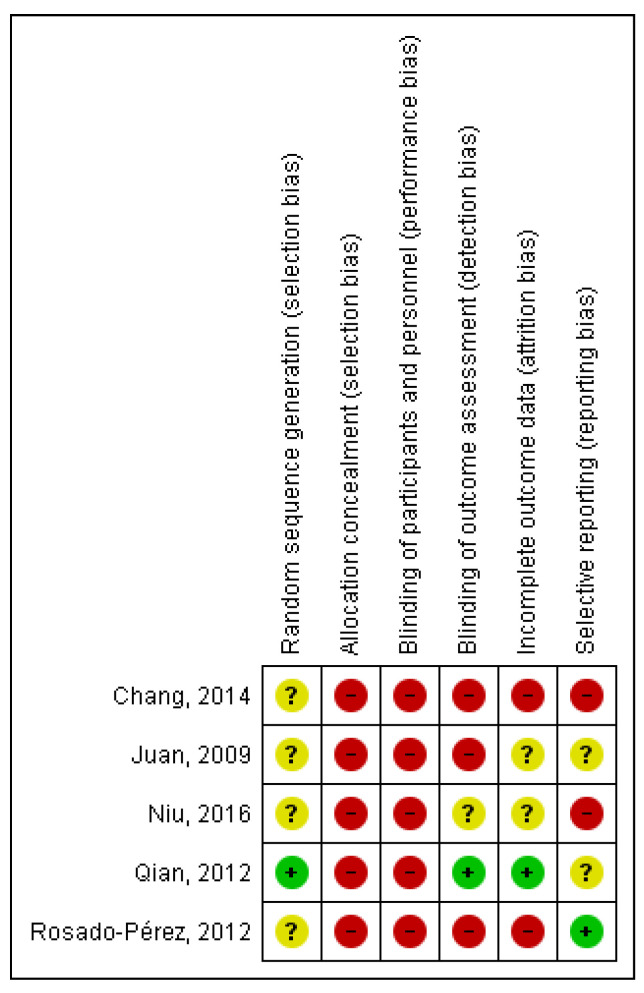
Summary diagram of risk of bias for randomized studies.

All five RCTs mention that the participants were randomized, but only one study mentions how the randomization sequence was generated (Figure 3). Due to the type of intervention, no study concealed allocation, and none blinded participants or staff. Three CRTs did not blind for outcome assessment. Two studies were at high risk of incomplete outcome data and two studies were at high risk of selective reporting bias.

The quality of the NRCTs included in this review was assessed using the ROBINS-I checklist. The pre-intervention, intervention, and post-intervention domains were assessed in each study based on key questions and detailed instructions in the ROBINS-I guidance manual (Table 3). All the studies assessed had at least one domain identified to be at serious risk of bias, resulting in an overall severe risk of bias for all studies.

## 4. Discussion

OxS is a dynamic process the evaluation of which depends on the determination of several markers; in the present review, we analyzed the effect of the practice of Tai Chi on those parameters reported consistently and comparably in the included studies.

### 4.1. Effect of Tai Chi on Antioxidant Enzyme SOD

Regarding the components of antioxidant defense, the result of the meta-analysis showed that there was an increase in the activity of the SOD enzyme in those who practiced Tai Chi; this is a finding reported in most of the studies included in this review [16,19,20,23], and even in studies excluded from the quantitative analysis [18,21]. We observed that this effect is maintained even when performing the sensitivity test, which shows the consistency of this result. SOD is a key enzyme in oxygen reduction reactions; it is responsible for dismuting the superoxide ion into hydrogen peroxide [32,33], and various interventions have reported that its activity is modified in the face of oxidative challenges such as exercise; however, the results are contradictory because, depending on the intensity and type of exercise, the response may be exhaustion or increase [34,35,36,37]. In this sense, the results of this meta-analysis are relevant, and they suggest that the practice of Tai Chi induces a sustained stimulus to increase the antioxidant response, since, regardless of the duration of the sessions, the effect of increasing activity is maintained, which is also consistent with the effects observed in the other markers analyzed.

### 4.2. Effect of Tai Chi on GPx and Gluthatione

Regarding the activity of glutathione peroxidase, even though in most of the studies included in this review the individual result showed an increase in its activity on Tai Chi practitioners [16,19], the global result of the quantitative analysis only shows a trend in favor of Tai Chi vs. sedentary behavior, which is maintained even after the sensitivity analysis. This trend was also observed in the global result of the quantitative analysis of glutathione concentration, a marker reported only in two studies, but whose result is consistent with the observation regarding GPx activity, since both parameters provide information on the functioning of the glutathione system, which is responsible for maintaining the intracellular redox state [33,38,39,40]. For both markers, the results suggest an increasing trend.

### 4.3. Effect of Tai Chi vs. Walking on GPx Activity

When performing the sub-analysis of comparison between walking and Tai Chi, an effect in favor of walking was observed, which coincides with the findings of other authors who point out an increase in this enzyme after performing interventions with walking or with higher-intensity aerobic activities [41,42,43]. The results suggest that the physical activity carried out promoted the increase in the activity of this enzyme both in those who practiced Tai Chi and in those who walked; however, the difference in the size of the observed effect may be due to the fact that walking, as an imminently aerobic activity, implied the generation of more RS, which demanded a greater antioxidant activity, in this case presumably provided by this enzyme.

### 4.4. Effect of Tai Chi on Enzyme Catalase 

Regarding the activity of the catalase, reported in two of the analyzed studies, we observed that the global result of the meta-analysis shows an increase in its activity in those who practiced Tai Chi compared to sedentary subjects, which is consistent with the previously explained findings.

The data analyzed up to this point allow us to suppose that the practice of Tai Chi has a positive effect on the expression and or activity of the mentioned endogenous enzymatic antioxidant components, whose function is to prevent the propagation of the oxidation of biomolecules [32,43].

Although these enzymes do not prevent the formation of RS, they prevent them from oxidizing cellular components since they catalyze their conversion into water [32,34]. The biochemical mechanism has been widely studied: SOD catalyzes the dismutation of the superoxide anion into hydrogen peroxide, which in turn is converted into water by the action of both GPx and catalase, hence these enzymes are considered part of the primary antioxidant system [44,45], and according to the results of this meta-analysis, they are susceptible to modification by providing an adequate stimulus, as the practice of Tai Chi appears to be [46]. 

### 4.5. Effect of Tai Chi on LPO and for 8-OH Guanosine

Regarding LPO, an indicator of oxidative damage to lipids, the global result of the meta-analysis showed that they are reduced in the subjects who practiced Tai Chi; this finding was consistent after the sensitivity analysis, and coincides with the result observed for 8-OH guanosine, a marker of oxidative damage to DNA, the levels of which were lower in those who practiced Tai Chi. These findings are congruent with the results obtained in the antioxidant markers because, when interpreting these data together, it is feasible to assume that the oxidative stimulus represented by Tai Chi had sufficient intensity to stimulate the antioxidant response so that it increased and the markers of oxidation decreased; this effect has been consistently associated with the practice of moderate-intensity exercise [41,47,48].

Two studies reported MDA as a marker of lipid damage, and although a downward trend was observed in those who practiced Tai Chi, this finding supports the results of the other oxidation markers.

### 4.6. Effect of Tai Chi vs. Walking on LPO

In the study wherein Tai Chi is compared to walking, a reduction in LPO in favor of Tai Chi was observed, which agrees with the previous indication regarding the aerobic nature of walking that possibly implied a greater oxidative challenge, to which it responded with an increase in the activity of GPx (also observed). This was, though, perhaps not enough to reduce the oxidative damage induced by RS caused by walking [41,49].

Regarding the study in which the parameters in saliva were reported, no difference was found; in this case, this may be due to the nature of the sample analyzed.

### 4.7. Effect of Tai Chi on TAS

Regarding the total serum antioxidant activity marker, the global result of the four studies included in the quantitative analysis did not show a difference between Tai Chi and sedentary behavior. This is possibly because endogenous and exogenous non-enzymatic antioxidants are determined with this marker, whose function is to prevent the propagation of damage by giving electrons to the RS, that is, they are secondary antioxidants. Considering that, according to the observed results, there was an increase in the enzymatic primary antioxidant response, it is also feasible to assume that an increase in the secondary antioxidant response was not necessary, hence no difference was observed; besides this, among these antioxidants, we found vitamins, as well as substances that depend on the diet and are not necessarily inducible [41,50].

### 4.8. Effect of Tai Chi vs. Yoga

Previous evidence suggests that yoga practice reduces OxS in patients with metabolic syndrome when compared to a dietary intervention [51]. In this review, we identified a study that showed no differences in oxidative stress markers between participants who practiced Tai Chi and yoga; however, the study did not show sufficient data to be included in the quantitative analysis.

Finally, the results of the quantitative analysis show that the practice of Tai Chi had an antioxidant effect, which is evident both in the increase in the activity of antioxidant enzymes and in the decrease in oxidation markers. In this sense, it has been recognized that the heterogeneity of the markers, techniques, and even samples analyzed makes it difficult to have cut-off values that allow the clinical interpretation of OxS parameters [52]; however, we consider that the consistency of these results allows us to point out the Tai Chi practice as a potentially beneficial activity at the clinical level given the various positive health effects it generates, including the antioxidant effect. These results can be explained considering the nature of Tai Chi, an activity of moderate intensity which combines elements such as deep breathing and transcendental meditation [9,10].

This antioxidant effect can be explained by the hormesis process, in which the sustained stimulation of RS generated by moderate exercise promotes the antioxidant response through an adaptive process [53,54,55,56]. Redox-sensitive signaling pathways use SR to transfer signals from the membrane to the nucleus; in this context, the main implicated proteins are MAPK and FNkB, which act synergistically to produce a controlled response. The proposed mechanism suggests that the RS generated during moderate physical activity act as the necessary signal for the activation of MAPK proteins (p38 and ERK1/ERK2), which in turn activate the transcription factor sensitive to the redox state, FNkB. This then migrates towards the nucleus, where it promotes the synthesis of several enzymes, such as MnSOD, iNOS, and glutamylcysteine synthetase (GCS) that have binding sites for this factor in the promoter region of its gene, which in general manifests as an increase in the antioxidant response to the moderate and sustained oxidative stimulus caused by this degree of physical activity [57,58,59].

However, the fact that there are multiple redox-sensitive binding sites in antioxidant genes suggests that the synergistic activation and interaction of several transcription factors is necessary to ensure the precision and safety of gene expression that results in an effective antioxidant response, which appears to be achieved through Tai Chi [58,59]. Likewise, it has been reported that regular exercise affects the activity of the nuclear factor erythroid 2-related factor 2 (Nrf2), as well as the downstream objectives of Nrf2 signaling. Nrf2 is pointed to as the major regulator of antioxidant defenses, since it regulates the expression of more than 200 cytoprotective genes. Recent evidence has indicated that Nrf2 signaling plays a central role in the beneficial effects of exercise mediated by oxidative stress. Regular exercise leads to the upregulation of endogenous antioxidant defenses as well as counteracting the harmful effects of RS [57,58,60].

Given the nature of Tai Chi, the antioxidant effect may be due not only to the hormetic process described, but also to the interaction between several mechanisms linked to meditation and diaphragmatic breathing. It has been shown that the practice of transcendental meditation (characteristic of Tai Chi) is associated with a better antioxidant response [61,62]; meanwhile, on the other hand, psychosocial stress is linked to an oxidative status [63]. Exposure to intrinsic or extrinsic stressors compromises the cellular redox homeostasis of the organism, resulting in a preponderance of oxidants. It has recently been reported that psychological stress leads to autonomic nervous system dysfunction, increased lipid peroxidation and oxidative stress, elevated expressions of inflammatory markers, and endothelial dysfunction. A growing body of evidence suggests a positive correlation between psychological stress and the increased production of free radicals and OxS, as well as the elevated release of stress hormones, blood pressure increase, proinflammatory activity in vascular tissue, and enhanced endothelial dysfunction [63,64,65,66,67]. By contrast, transcendental meditation has been linked to beneficial effects, and clinical research studies have demonstrated the favorable effects of mindfulness-based meditation practices in decreasing cardiovascular risk factors such as hypertension, lipid peroxidation, hyperlipidemia, and atherosclerosis [63,64]. Some mechanisms have been proposed, including the preservation of the autonomic system’s nervous homeostasis and the regulation of cytokines [68]. As regards diaphragmatic breathing, increases in antioxidant defense status and melatonin levels have been reported, with concomitant reductions in cortisol levels [69]. It has been suggested that the varied beneficial effects of Tai Chi are due to a combination of the mentioned mechanisms, which, coupled with the fact that its practice does not involve risks, makes it an ideal activity for older adults [61].

In this regard, the results of this meta-analysis suggest an antioxidant effect associated with the practice of this activity; however, some limitations need to be considered when interpreting the results of this study. First, the meta-analysis results for most of the OxSM exhibit significant heterogeneity, which may be due to differences in the types of study as well as to differences in the duration and the number of times a week the intervention with Tai Chi was performed. Second, all the included studies only reported results based on per protocol analysis, and this fact could bias the results due to the loss of participants, especially in the studies where the comparison was made with sedentary behavior. Third, most of the included studies did not adequately describe the population at baseline, and therefore it is not possible to know whether the groups were balanced in variables such as age and sex before starting the intervention. Finally, the evaluation of publication bias could not be carried out because none of the comparisons identified more than 10 articles.

## 5. Conclusions

Regular Tai Chi practice increases the levels of the antioxidant enzymes superoxide dismutase and catalase, as well as reducing the levels of lipoperoxides when compared to sedentary behavior. More studies are necessary to determine the effect of Tai Chi on oxidative stress markers when compared to other physical activities such as walking or yoga.

## Figures and Tables

**Table 1:** Characteristics of the included studies (n = 10).

Autor, Year	Study Design	Participants	Tai Chi	N	Comparator	N	Follow-up, m	Oxidative Stress Markers	Included in Quantitative Synthesis
Juan, 2009 [16]	RCT	Women younger than 55 years old.	Daily, two times a day in 30 min sessions.	30	Sedentary behavior.	30	3	SOD, GPx, CAT, MDA, GSH	Yes
Goon, 2009 [21]	NRCT	Adults over 45 years.	Twice a week in 1 h sessions.	15	Sedentary behavior.	17	6, 12	SOD, GPx, CAT, MDA	No
Montalvo-Olvera, 2011 [22]	NRTC	Older adults, average age of 65 years.	Five times a week in 50 min sessions.	18	Sedentary behavior.	18	12	SOD, GPx, LPO, TAS	Yes
Qian, 2012 [17]	RCT	Postmenopausal women with at least two years after menopause with a diagnosis of osteopenia.	Three times a week in 1 h sessions. In addition, they consumed 250 mg of medicinal starch twice a day daily.	37	Sedentary behavior. In addition, they consumed 250 mg of medicinal starch twice a day daily.	37	1, 3, 6	Urinary 8-OHdG	Yes
Rosado-Pérez, 2012 [20]	RCT	Older adults in an urban community.	Three times a week in 1 h sessions.	32	Sedentary behavior.	23	6	SOD, GPx, LPO, TAS	Yes
Rosado-Pérez, 2013 [23]	NRCT	Older adults with an age between 60 and 74 years.	Daily in 1 h sessions.	32	Sedentary behavior. Daily 1 h walking sessions.	40 43	6	SOD, GPx, LPO, TAS	Yes
Chang, 2014 [18]	RCT	Young female undergraduate volunteers, with an average age of 18 years.	Three times a week in 40 min sessions	12	Sedentary behavior. Yoga three times a week in 40 min sessions.	12 12	2 ½	SOD, GPx, MDA	No
Mendoza-Núñez, 2014 [24]	NRCT	Sedentary older adults aged between 60 and 74 years.	Five times a week in 1 h sessions	24	Sedentary behavior.	25	6	Salival: SOD, LPO, TAS	Yes
Niu, 2016 [19]	RCT	Adults aged 40–45 years.	Daily in 1 h sessions.	25	Sedentary behavior.	25	2, 4, 6	SOD, GPx, CAT, MDA, GSH	Yes
Mendoza-Núñez, 2018 [25]	NRCT	Mexican mestizo older adults aged 60–74 years.	Five times a week for 50 min sessions.	48	Sedentary behavior.	37	6	SOD, GPx, LPO, TAS	Yes

CAT, catalase; GSH, glutathione; GPx, glutathione peroxidase; MDA, malondialdehyde; LPO, lipoperoxides; m, months; NRCT, non-randomized clinical trial; RCT, randomized clinical trial; SOD, superoxide dismutase; TAS, total antioxidant status; 8-OHdG, 8-hydroxy-2′-deoxyguanosine.

**Table 2:** Pooled mean difference in oxidative stress markers.

Oxidative Marker	No. of Studies	MD [95%CI], Participants, DerSimonian and Laird MODEL	Heterogeneity	
			I^2^ (%)	*p* Value
Tai Chi vs. Sedentary behavior				
Glutathione peroxidase, U/Ml	6	2.40 [−0.48 to 5.27], 344	92	<0.00001
Total antioxidant status, mmol/L	4	0.12 [−0.03 to 0.27], 222	88	<0.0001
Catalase, U/Ml	2	15.63 [4.05 to 27.22], 110	79	0.03
Glutathione, mg/L	2	77.79 [−11.03 to 166.61],110	97	<0.00001
Lipoperoxides, µmol/L	4	−0.02 [−0.04 to −0.00], 234	0	0.53
Malondialdehyde, nmol/Ml	2	−2.71 [−5.45 to 0.04], 110	96	<0.00001
Saliva superoxide dismutase, IU/mL	1	0.46 [−3.35 to 4.27], 49	NA	NA
Saliva total antioxidant status, mmol/L	1	0.08 [−0.81 to 0.97], 49	NA	NA
Saliva lipoperoxides, µmol/L	1	0.06 [−0.19 to 0.31], 49	NA	NA
Urinary 8-OHdG, ng/mg creatinine	1	−35.70 [−53.09 to −18.31], 74	NA	NA
Tai Chi vs. Walking				
Superoxide dismutase, U/Ml	1	1.0 [−3.16 to 5.16], 74	NA	NA
Glutathione peroxidase, U/Ml	1	−3.19 [−0.10 to −0.04], 74	NA	NA
Lipoperoxides, µmol/L	1	−0.07 [−0.10 to −0.04], 74	NA	NA
Total antioxidant capacity, mmol/L	1	0.03 [−0.04 to 0.10], 74	NA	NA

Abbreviations: 8-OHdG, 8-hydroxy-2′-deoxyguanosine; NA, not applicable; MD, mean difference.

**Table 3:** Risk of bias in non-randomized interventions (ROBINS-I tool).

	Pre-Intervention		At Intervention	Post-Intervention				
Author	Bias Due to Confounding	Bias in Selection of Participants into the Study	Bias in Classification of Interventions	Bias Due to Deviations from Intended Intervention	Bias Due to Missing Data	Bias in Measurement of Outcomes	Bias in Selection of the Reported Result	Overall Risk of Bias
Goon, 2009	L	M	L	S	S	M	S	S
Montalvo-Olvera, 2011	M	S	L	L	L	M	S	S
Rosado-Pérez, 2013	M	S	L	S	S	M	S	S
Mendoza-Núñez, 2014	M	S	L	S	S	M	S	S
Mendoza-Núñez, 2018	M	S	L	S	L	M	S	S

Abbreviations: L, low risk of bias; M, moderate risk of bias; N, no information; S, serious risk of bias.

## Data Availability

Not applicable.

## Supplementary Materials

The following are available online at https://www.mdpi.com/article/10.3390/ijerph18073458/s1, Table S1: Characteristics of the excluded studies (n = 6).

## References

[1] Checa J., Aran J.M. (2020). Reactive Oxygen Species: Drivers of Physiological and Pathological Processes. Inflamm. Res..

[2] Phaniendra A., Jestadi D.B., Periyasamy L. (2015). Free radicals: Properties, sources, targets, and their implication in various diseases. Indian. J. Clin. Biochem..

[3] Rosado-Pérez J., Aguiñiga-Sánchez I., Arista-Ugalde T.L., Santiago-Osorio E., Mendoza-Núñez V.M. (2018). The Biological Significance of Oxidative Stress, Effects of Fruits as Natural Edible Antioxidants. Curr. Pharm. Des..

[4] Liguori I., Russo G., Curcio F., Bulli G., Aran L., Della-Morte D., Gargiulo G., Testa G., Cacciatore F., Bonaduce D. (2018). Oxidative stress, aging, and diseases. Clin. Interv. Aging.

[5] Reiter R.J., Mayo J.C., Tan D., Sainz R.M., Alatorre-Jimenez M., Qin L. (2016). Melatonin as an antioxidant: Under promises but over delivers. J. Pineal Res..

[6] Serafini M., Peluso I. (2016). Functional Foods for Health: The Interrelated Antioxidant and Anti-Inflammatory Role of Fruits, Vegetables, Herbs, Spices and Cocoa in Humans. Curr. Pharm. Des..

[7] Simioni C., Zauli G., Martelli A.M., Vitale M., Sacchetti G., Gonelli A., Neri L.M. (2018). Oxidative stress: Role of physical exercise and antioxidant nutraceuticals in adulthood and aging. Oncotarget.

[8] Solloway M.R., Taylor S.L., Shekelle P.G., Miake-Lye I.M., Beroes J.M., Shanman R.M., Hempe S. (2016). An evidence map of the effect of Tai Chi on health outcomes. Syst. Rev..

[9] Yang G.Y., Wang L.Q., Ren J., Zhang Y., Li M.-L., Zhu Y.T., Luo J., Cheng Y.G., Li W.Y., Wayne P.M. (2015). Evidence base of clinical studies on Tai Chi: A bibliometric analysis. PLoS ONE.

[10] Miller S.M., Hui-Lio C., Taylor-Piliae R.E. (2020). Health Benefits of Tai Chi Exercise: A Guide for Nurses. Nurs. Clin. N. Am..

[11] Klein P.J., Baumgarden J., Schneider R. (2019). Qigong and Tai Chi as Therapeutic Exercise: Survey of Systematic Reviews and Meta-Analyses Addressing Physical Health Conditions. Altern. Ther. Health Med..

[12] Wang F., Lee E.K.O., Wu T., Benson H., Fricchione G., Wang W., Yeung A.S. (2014). The effects of tai chi on depression, anxiety, and psychological well-being: A systematic review and meta-analysis. Int. J. Behav. Med..

[13] Higgins J.P.T., Altman D.G., Gøtzsche P.C., Jüni P., Moher D., Oxman A.D., Savović J., Schulz K.F., Weeks L., Sterne J.A.C. (2011). The Cochrane Collaboration’s tool for assessing risk of bias in randomised trials. BMJ.

[14] Sterne J.A., Hernán M.A., Reeves B.C., Savović J., Berkman N.D., Viswanathan M., Henry D., Altman D.G., Ansari M.T., Boutron I. (2016). ROBINS-I: A tool for assessing risk of bias in non-randomised studies of interventions. BMJ.

[15] The Nordic Cochrane Centre Review Manager (RevMan). http://tech.cochrane.org/revman/download.

[16] Juan J., YingJie G., AiJun N. (2009). Extraction, characterization of Angelica sinensis polysaccharides and modulatory effect of the polysaccharides and Tai Chi exercise on oxidative injury in middle-aged women subjects. Carbohydr. Polym..

[17] Qian G., Xue K., Tang L., Wang F., Song X., Chyu M.C., Pence B.C., Shen C.L., Wang J.S. (2012). Mitigation of Oxidative Damage by Green Tea Polyphenols and Tai Chi Exercise in Postmenopausal Women with Osteopenia. PLoS ONE.

[18] Chang T.C. (2014). The effect of short term yoga and Tai-Chi education exercise on antioxidant capacity and oxidative stress measures. Stud. Ethno Med..

[19] Niu A. (2016). Effect of “Tai Chi” exercise on antioxidant enzymes activities and immunity function in middle-aged participants. African. J. Tradit. Complement. Altern. Med..

[20] Rosado-Pérez J., Santiago-Osorio E., Ortiz R., Mendoza-Núñez V.M. (2012). Tai chi diminishes oxidative stress in mexican older adults. J. Nutr. Heath Aging.

[21] Goon J.A., Noor Aini A.H., Musalmah M., Yasmin Anum M.Y., Wan Nazaimoon W.M., Wan Ngah W.Z. (2009). Effect of Tai Chi exercise on DNA damage, antioxidant enzymes, and oxidative stress in middle-age adults. J. Phys. Act. Health.

[22] Montalvo-Olvera J. (2011). Efecto del Tai Chi vs. Suplementos Antioxidantes Para el Control del Estrés Oxidativo.

[23] Rosado-Pérez J., Ortiz R., Santiago-Osorio E., Mendoza-Núñez V.M. (2013). Effect of Tai Chi versus walking on oxidative stress in Mexican older adults. Oxid. Med. Cell. Longev..

[24] Mendoza-Núñez V.M., Hernández-Monjaraz B., Santiago-Osorio E., Betancourt-Rule J.M., Ruiz-Ramos M. (2014). Tai chi exercise increases SOD activity and total antioxidant status in saliva and is linked to an improvement of periodontal disease in the elderly. Oxid. Med. Cell. Longev..

[25] Mendoza-Núñez V.M., Arista-Ugalde T.L., Rosado-Pérez J., Ruiz-Ramos M., Santiago-Osorio E. (2018). Hypoglycemic and antioxidant effect of Tai Chi exercise training in older adults with metabolic syndrome. Clin. Interv. Aging.

[26] Palasuwan A., Margaritis I., Soogarun S., Rousseau A.S. (2011). Dietary intakes and antioxidant status in mind-body exercising pre- and postmenopausal women. J. Nutr. Health Aging.

[27] Palasuwan A., Suksom D., Margaritis I., Soogarun S., Rousseau A.S. (2011). Effects of tai chi training on antioxidant capacity in pre- and postmenopausal women. J. Aging Res..

[28] Di Nardo M., Gibson J.M., Siminerio L., Morell A.R., Lee E.S. (2012). Complementary and alternative medicine in diabetes care. Curr. Diab. Rep..

[29] Huang X., Eungpinichpong W., Silsirivanit A., Nakmareong S., Wu X.H. (2014). Tai chi improves oxidative stress response and DNA damage/repair in young sedentary females. J. Phys. Ther. Sci..

[30] Kasim N.F., Aldred S., Veldhuijzenvan J., Zanten Y. (2017). Acute effect of Thai Chi on marker of oxidative stress and flow-mediated dilation among healthy young and elderly volunteers. Free Radic. Biol. Med..

[31] Yu Y., Ga Q., Xia W., Zhang L., Hu Z., Wu X., Jia X. (2018). Association between Physical Exercise and Biomarkers of Oxidative Stress among Middle-Aged and Elderly Community Residents with Essential Hypertension in China. Biomed. Res. Int..

[32] He L., He T., Farrar S., Ji L., Liu T., Ma X. (2017). Antioxidants Maintain Cellular Redox Homeostasis by Elimination of Reactive Oxygen Species. Cell. Physiol. Biochem..

[33] Imlay J.A. (2008). Cellular defenses against superoxide and hydrogen peroxide. Annu. Rev. Biochem..

[34] Bouzid M.A., Filaire E., Matran R., Robin S., Fabre C. (2018). Lifelong Voluntary Exercise Modulates Age-Related Changes in Oxidative Stress. Int. J. Sports Med..

[35] Bouzid M.A., Hammouda O., Matran R., Robin S., Fabre C. (2015). Influence of physical fitness on antioxidant activity and malondialdehyde level in healthy older adults. Appl. Physiol. Nutr. Metab..

[36] Kostka T., Drai J., Berthouze S.E., Lacour J.R., Bonnefoy M. (1998). Physical activity, fitness and integrated antioxidant system in healthy active elderly women. Int. J. Sports Med..

[37] Gagnon D.D., Dorman S., Ritchie S., Mutt S.J., Stenbäck V., Walkowiak J.W., Herzig K. (2019). Multi-Day Prolonged Low- to Moderate-Intensity Endurance Exercise Mimics Training Improvements in Metabolic and Oxidative Profiles Without Concurrent Chromosomal Changes in Healthy Adults. Front. Physiol..

[38] Deponte M. (2013). Glutathione catalysis and the reaction mechanisms of glutathione-dependent enzymes. Biochim. Biophys. Acta.

[39] Lu S.C. (2013). Glutathione synthesis. Biochim. Biophys. Acta.

[40] Teskey G., Abrahem R., Cao R., Gyurjian K., Islamoglu H., Lucero M., Martinez A., Paredes E., Salaiz O., Robinson B. (2018). Glutathione as a Marker for Human Disease. Adv. Clin. Chem..

[41] Karolkiewicz J., Michalak E., Pospieszna B., Deskur-Smielecka E., Nowak A., Łucja Pilaczyńska-Szcześniak L. (2009). Response of oxidative stress markers and antioxidant parameters to an 8-week aerobic physical activity program in healthy, postmenopausal women. Arch. Gerontol. Geriatr..

[42] Karolkiewicz J., Szczêsniak L., Deskur-Smielecka E., Nowak A., Stemplewski R., Szeklicki R. (2003). Oxidative stress and antioxidant defense system in healthy, elderly men: Relationship to physical activity. Aging Male.

[43] Sachdev S., Davies K.J.A. (2008). Production, detection, and adaptive responses to free radicals in exercise. Free Radic. Biol. Med..

[44] Galle F.A., Martella D., Bresciani G. (2018). Antioxidant and anti-inflammatory modulation of exercise during aging. Rev. Esp. Geriatr. Gerontol..

[45] Li R., Jia Z., Trush M.A. (2016). Defining ROS in Biology and Medicine. React. Oxyg. Species.

[46] Valavanidis A., Vlachogianni T., Fiotakis C. (2009). 8-hydroxy-2’-deoxyguanosine (8-OHdG): A critical biomarker of oxidative stress and carcinogenesis. J. Environ. Sci. Health. C.

[47] Goto C., Higashi Y., Kimura M., Noma K., Hara K., Nakagawa K., Kawamura M., Chayama K., Yoshizumi M., Nara I. (2003). Effect of different intensities of exercise on endothelium-dependent vasodilation in humans: Role of endothelium-dependent nitric oxide and oxidative stress. Circulation.

[48] Gomez-Cabrera M.C., Salvador-Pascual A., Cabo H., Ferrando B., Viña J. (2015). Redox modulation of mitochondriogenesis in exercise. Does antioxidant supplementation blunt the benefits of exercise training?. Free Radic. Biol. Med..

[49] Busquets-Cortés C., Capó X., Bibiloni M.M., Martorell M., Ferrer M.D., Argelich E., Bouzas C., Carreres S., Tur J.A., Pons A. (2018). Peripheral Blood Mononuclear Cells Antioxidant Adaptations to Regular Physical Activity in Elderly People. Nutrients.

[50] Huang D., Ou B., Prior R.L. (2005). The chemistry behind antioxidant capacity assays. J. Agric. Food Chem..

[51] Yadav R., Yadav R.K., Khadgawat R., Pandey R.M. (2019). Comparative efficacy of a 12 week yoga-based lifestyle intervention and dietary intervention on adipokines, inflammation, and oxidative stress in adults with metabolic syndrome: A randomized controlled trial. Transl. Behav. Med..

[52] Sánchez-Rodríguez M.A., Mendoza-Núñez V.M. (2019). Oxidative Stress Indexes for Diagnosis of Health or Disease in Humans. Oxid. Med. Cell. Longev..

[53] Ji L.L., Kang C., Zhang Y. (2016). Exercise-induced hormesis and skeletal muscle health. Free Radic. Biol. Med..

[54] Powers S.K., Radak Z., Ji L.L. (2016). Exercise-induced oxidative stress: Past, present and future. J. Physiol..

[55] Bouzid M.A., Filaire E., McCall A., Fabre C. (2015). Radical Oxygen Species, Exercise and Aging: An Update. Sports Med..

[56] Powers S.K., Jackson M.J. (2008). Exercise-induced oxidative stress: Cellular mechanisms and impact on muscle force production. Physiol. Rev..

[57] Di Meo S., Napolitano G., Venditti P. (2019). Mediators of Physical Activity Protection against ROS-Linked Skeletal Muscle Damage. Int. J. Mol. Sci..

[58] Ji L.L. (2015). Redox signaling in skeletal muscle: Role of aging and exercise. Adv. Physiol. Educ..

[59] Scandalios J.G. (2005). Oxidative stress: Molecular perception and transduction of signals triggering antioxidant gene defenses. Braz. J. Med. Biol. Res..

[60] Done A.J., Traustadóttir T. (2016). Nrf2 mediates redox adaptations to exercise. Redox. Biol..

[61] Yan J.H., Downing J.H. (1998). Tai Chi: An alternative exercise form for seniors. J. Aging Phys. Act..

[62] Kim D., Moon Y., Kim H., Jung J., Park H., Suh H., Kim Y., Song D. (2005). Effect of Zen Meditation on serum nitric oxide activity and lipid peroxidation. Prog. Neuropsychopharmacol. Biol. Psychiatry.

[63] Wang L., Muxin G., Nishida H., Shirakawa C., Sato S., Konishi T. (2007). Psychological stress-induced oxidative stress as a model of sub-healthy condition and the effect of TCM. Evid. Based Complement. Alternat. Med..

[64] Amarasekera A.T., Chang D. (2019). Buddhist meditation for vascular function: A narrative review. Integr. Med. Res..

[65] Siegrist J., Sies H. (2017). Disturbed Redox Homeostasis in Oxidative Distress: A Molecular Link From Chronic Psychosocial Work Stress to Coronary Heart Disease?. Circ. Res..

[66] Cohen B.E., Edmondson D., Kronish I.M. (2015). State of the Art Review: Depression, Stress, Anxiety, and Cardiovascular Disease. Am. J. Hypertens..

[67] Xue T., Li H., Wang M., Shi Y., Shi K., Cheng Y., Cui D. (2018). Mindfulness meditation improves metabolic profiles in healthy and depressive participants. CNS Neurosci. Ther..

[68] Hui S., Meijuan C., Donghong C. (2020). Biological mechanism study of meditation and its application in mental disorders. Gen. Psychiatr..

[69] Martarelli D., Cocchioni M., Scuri S., Pompei P. (2011). Diaphragmatic breathing reduces exercise-induced oxidative stress. Evid. Based Complement. Altern. Med..

