# Supplementary material for: Effect of Tai Chi on Markers of Oxidative Stress: Systematic Review and Meta-Analysis
Source: Int J Environ Res Public Health. 2021 Mar 26;18(7):3458. doi: 10.3390/ijerph18073458 (PMC8037964; doi:10.3390/ijerph18073458)
Supplement: Supplementary file 1 [file ijerph-18-03458-s001.pdf]

Table S1. Characteristics of the excluded studies (n = 6).

Reference	Reasons for exclusion
Palasuwan, 2011a[26]	Cross-sectional study
Palasuwan, 2011b[27]	Non-comparative study
DiNardo, 2012[28]	Narrative review
Huang, 2014[29]	Non-comparative study
Kasim, 2017[30]	Shortest follow-up time
Yu Y, 2018[31]	Cross-sectional study
